# Comparison of placenta samples with contamination controls does not provide evidence for a distinct placenta microbiota

**DOI:** 10.1186/s40168-016-0172-3

**Published:** 2016-06-23

**Authors:** Abigail P. Lauder, Aoife M. Roche, Scott Sherrill-Mix, Aubrey Bailey, Alice L. Laughlin, Kyle Bittinger, Rita Leite, Michal A. Elovitz, Samuel Parry, Frederic D. Bushman

**Affiliations:** Department of Microbiology, Perelman School of Medicine at the University of Pennsylvania, 3610 Hamilton Walk, Philadelphia, PA 19104-6076 USA; Department of Obstetrics and Gynecology, Maternal and Child Health Research Program, Perelman School of Medicine at the University of Pennsylvania, 3400 Spruce Street, Philadelphia, PA 19104 USA

**Keywords:** Placenta, Microbiome, 16S rRNA gene, Low biomass samples, Reagent contamination

## Abstract

**Background:**

Recent studies have suggested that bacteria associated with the placenta—a “placental microbiome”—may be important in reproductive health and disease. However, a challenge in working with specimens with low bacterial biomass, such as placental samples, is that some or all of the bacterial DNA may derive from contamination in dust or commercial reagents. To investigate this, we compared placental samples from healthy deliveries to a matched set of contamination controls, as well as to oral and vaginal samples from the same women.

**Results:**

We quantified total 16S rRNA gene copies using quantitative PCR and found that placental samples and negative controls contained low and indistinguishable copy numbers. Oral and vaginal swab samples, in contrast, showed higher copy numbers. We carried out 16S rRNA gene sequencing and community analysis and found no separation between communities from placental samples and contamination controls, though oral and vaginal samples showed characteristic, distinctive composition. Two different DNA purification methods were compared with similar conclusions, though the composition of the contamination background differed. Authentically present microbiota should yield mostly similar results regardless of the purification method used—this was seen for oral samples, but no placental bacterial lineages were (1) shared between extraction methods, (2) present at >1 % of the total, and (3) present at greater abundance in placental samples than contamination controls.

**Conclusions:**

We conclude that for this sample set, using the methods described, we could not distinguish between placental samples and contamination introduced during DNA purification.

**Electronic supplementary material:**

The online version of this article (doi:10.1186/s40168-016-0172-3) contains supplementary material, which is available to authorized users.

## Background

The placenta is a known reservoir for microbes that can lead to adverse pregnancy outcomes (reviewed in [1] and [2]). However, the concept of commensal microbial communities that could become dysbiotic and induce placental dysfunction, leading to adverse outcome, has not been established. Several recent studies introduced the idea that the healthy human placenta harbors a unique low-abundance microbiome [3–6]. Tracking of lineages enriched in the placental samples suggested a possible oral origin [3]. These data were intriguing, potentially paralleling published imaging studies suggesting detection of intracellular bacteria in the basal plate of placentas at the maternal-fetal interface [7, 8]. However, studies of low biomass samples such as placenta are known to be exceedingly difficult. When low amounts of bacteria are present in a sample, contaminating sources of bacterial DNA can predominate, such that sequences recovered may contain mostly contamination from dust, commercial reagents, or other sources [9, 10]. In the report of a commensal placental microbiome [3], only a partial set of control studies was reported, leaving the possible contribution of contamination unclear.

Given our interest in microbial infection and preterm birth, we sought to carry out a comprehensive study of placental samples to evaluate possible microbial colonization and to provide a platform for future research. Because our focus was on issues of sampling and contamination, we analyzed an extensive set of matched experimental and control samples (7 samples per subject, plus additional contamination controls) for six subjects. Placental tissue was harvested from the basal plate and the fetal side of the placenta using a uniform protocol. These placental samples were compared to blank swabs waved in air in the clinical laboratory where placental samples were processed, unused sterile swabs, and DNA purifications with no added starting material. We also compared oral and vaginal samples from the same women obtained at admission to the hospital for delivery. We used real-time quantitative polymerase chain reaction (qPCR) for absolute quantification of bacterial sequence abundance and 16S rRNA gene tag sequencing to quantify community composition. Two different methods for DNA purification were compared, because low-level contamination has been reported to derive from commercial kits [9–11]. In both assays (qPCR and 16S rRNA gene tag sequencing), placental samples could not be distinguished from background contamination controls.

## Results

### Sampling strategy

Six women were selected for study with uncomplicated singleton pregnancies at term in spontaneous labor (regular contractions, cervical dilation) or following spontaneous rupture of membranes. Demographic and clinical characteristics of participants are listed in Additional file 1: Table S1. Sample types collected are listed in Table 1. Placental samples were isolated as 0.5 × 0.5 × 0.5 cm cuboidal sections from internal structures within the placenta (maternal and fetal sides) to eliminate surface contamination. Maternal saliva samples and vaginal swab samples were also collected by study personnel from participants at admission to the hospital preceding delivery. Analysis of paired samples from the same patient is a notable difference from [3], where non-placental samples (vaginal/oral) were from historical controls.

**Table 1:** Sample types studied

Sample	Collection protocol/comments
Air swab	Swab of the air from the clinical laboratory where the placental samples were obtained
Sterile swab	Unopened sterile swab
Extraction blank	Blank tubes for possible extraction/reagent contaminants
Placenta (MS)	Basal plate biopsy obtained after removal of placental surface
Placenta (FS)	Placental biopsy obtained after removal of placental surface
Saliva	Collected in sterile 50-mL conical tube
Vaginal swab	Swab inserted into maternal vagina for 30 s

Three types of negative controls were collected. Following each delivery, a swab was waved in the air in the clinical laboratory where the placental biopsies were carried out (adjacent to the labor and delivery rooms), then sealed in a closed container (designated “air swab”). Unused sterile swabs were also collected (“sterile swab”). Thirdly, samples containing the purification reagents only were purified by each method to document bacterial sequences introduced during downstream sample processing (“extraction blank”).

DNA was then purified from each specimen. Two different kits were compared, the STRATEC PSP Spin Stool DNA Plus Kit (henceforth “PSP”) and the MO BIO PowerSoil DNA Isolation Kit (abbreviated “MO BIO”), both of which have been used extensively in microbiome research, and the second of which was used by Aagaard et al. [3], which proposed the existence of a commensal microbiota. An additional reason for comparing these two methods is to identify reagent contamination, because contaminating 16S rRNA gene sequences introduced by DNA purification kits tend to differ by kit [9, 10].

### Analysis of total 16S rRNA gene copies using quantitative PCR

**Fig. 1:**
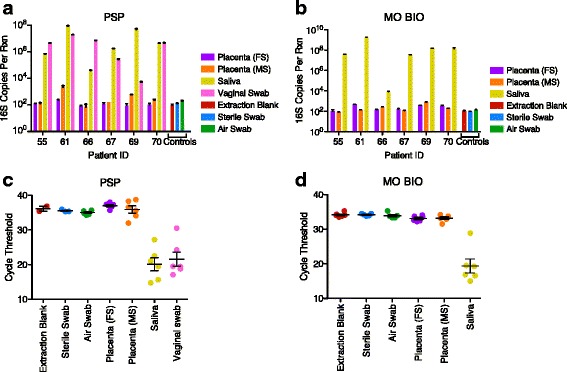
Quantitative PCR analysis of 16S rRNA gene copy numbers. 16S rRNA gene copies per reaction were quantified in the six study subjects using (**a**) PSP extraction method and (**b**) MO BIO extraction method. Fetal side (*FS*), maternal side (*MS*). **c**, **d** Comparison of mean cycle threshold values for 16S rRNA gene qPCR. Limit of detection is 38.29 (PSP) and 34.04 (MO BIO). All data sets were analyzed by Kruskal-Wallis with Dunn’s post test. Comparison of any pair of placental samples to controls yielded *p* > 0.05

Quantification of 16S rRNA gene copies by quantitative PCR is shown in Fig. 1a, b. For qPCR, equal volumes of the purified DNA of all samples were used in the assay. In this and subsequent figures, results for DNA samples prepared using the two purification kits are shown side by side. For the PSP kit, the mean 16S rRNA gene copies in the saliva samples were 2.3 × 10^7^ ± 1.42 × 10^7^ SEM (standard error of the mean). Mean copy number in the vaginal samples was 5.97 × 10^6^ ± 2.92 × 10^6^ SEM. Results for placental samples were much lower, with mean copy number for maternal side (MS) at 5.72 × 10^2^ ± 3.48 × 10^2^ SEM and fetal side (FS) at 1.24 × 10^2^ ± 2.1 × 10^1^ SEM. For controls, the mean copy number was between 9.7 × 10^1^ ± 4.1 × 10^1^ and 1.93 × 10^2^ ± 3.07 × 10^1^ SEM. For the MO BIO kit, mean copy number for the saliva samples was high, 3.69 × 10^8^ ± 2.98 × 10^8^ SEM, while mean copy numbers for the placental samples were again much lower and indistinguishable from contamination controls; maternal side placenta was 2.56 × 10^2^ ± 1.05 × 10^2^ SEM, fetal side was 2.61 × 10^2^ ± 6.3 × 10^1^ SEM, and for controls mean copy numbers were between 8.77 × 10^1^ ± 1.1 × 10^1^ SEM and 1.29 × 10^2^ ± 2.87 × 10^1^ SEM.

For statistical analysis, we compared the cycle of threshold values for all sample sets, because this represents the rawest form of the data (Fig. 1c, d; amplification curves in Additional file 2: Figure S1). The cycle of threshold indicates the value at which exponential increase in the fluorescence signal initiated, so larger values indicate lower numbers of starting templates. For tests of samples purified using either method, there was no significant difference between median values for any pair of placental and control samples (*p* > 0.05; Kruskal-Wallis test with Dunn’s post test). In comparisons without correction for multiple comparisons, one pair showed a difference in the direction of the negative control air swab amplifying at lower cycle of threshold (more template copies) than the fetal side placental sample (Fig. 1c).

Using a Bayesian approach, it is possible to interrogate these data further (Additional file 3: Figure S2). A posterior probability distribution was calculated for the difference in mean cycle numbers between air swabs and all other sample types. Based on the posterior probability distribution, we calculated that for both placental samples extracted by either of the methods, comparison to air swabs yielded *p* > 0.05 for the probability of greater abundance of 16S rRNA gene DNA in the placental samples, as was seen with conventional hypothesis testing. Further, we calculate the probability that the pooled placental samples contain 10-fold or more 16S rRNA gene copies than air swabs as *p* = 0.00004 for PSP and *p* = 0.004 for MO BIO. In addition, the analysis shows that there is a 95 % probability that the placental samples have <3.5× more DNA than air swabs in MO BIO and <0.8× DNA for PSP. For comparison, the vaginal or oral swabs have ~30,000-fold more DNA than controls when compared by this method.

In summary, all placental and negative control samples were at the extreme low end of the range detectable by qPCR (Additional file 2: Figure S1A–C). For both purification methods, we conclude that placental and control samples showed low and indistinguishable numbers of 16S rRNA gene copies.

### Analysis of bacterial community structure using deep sequencing

Samples were next compared based on the proportions of bacterial lineages in each specimen using deep sequencing, investigating the hypothesis that the types of bacteria present in placental and control samples differed. Samples were pooled for sequencing by adding equal volumes of the purified DNA from placental and control samples and equal masses of DNA for the higher biomass oral and vaginal samples.

**Fig. 2:**
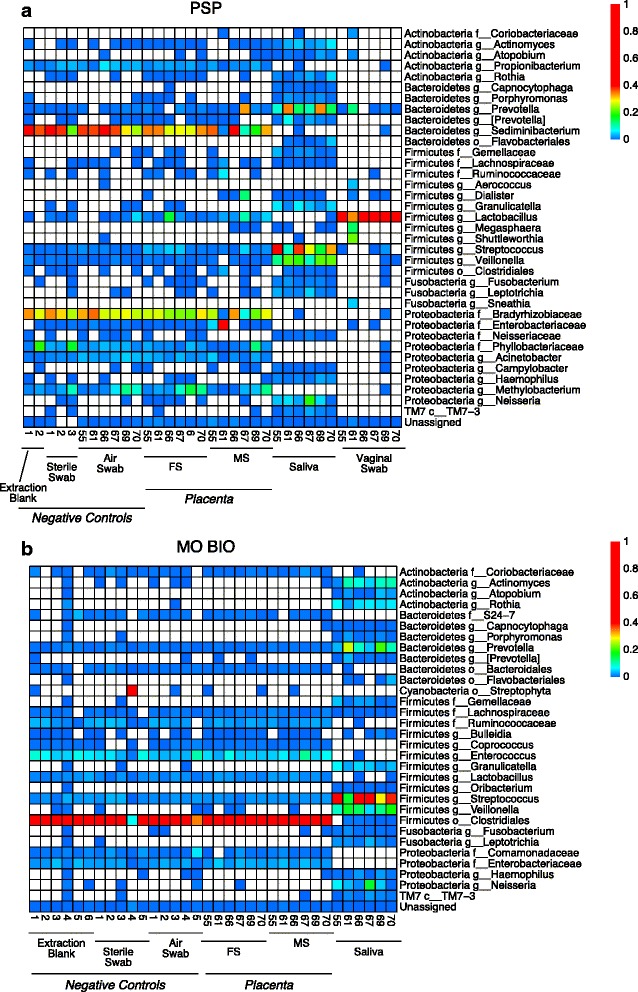
Heat maps illustrating the major bacterial lineages detected in each sample. Heat maps of major bacterial lineages for (**a**) PSP- and (**b**) MO BIO-extracted samples. *Columns* indicate the patient ID and sample types, and *rows* indicate the OTU detected in sequencing. Fetal side (*FS*), maternal side (*MS*), family (*f*), genus (*g*), order (*o*)

DNA samples were PCR amplified using bar-coded primers flanking the V1V2 region of the 16S rRNA gene, and samples were sequenced using the Illumina method (Fig. 2a, b). The V1V2 region is useful with low biomass samples because the shorter length allows more efficient amplification of rare template sequences, as shown, for example, in studies of bronchoalveolar lavage samples [11–15], and V1V2 has been used extensively in previous work [16–18]. An alternative approach is to use shotgun metagenomic sequencing to characterize all DNA present in a sample [3]—however, this has the disadvantage for placental samples that the great majority of the sequencing effort is expended resequencing human DNA and so provides little additional information at a much higher cost when only a characterization of bacterial taxa present is desired. Each sample returned an average of 59,198 (PSP extraction) and 25,044 (MO BIO extraction) 16S rRNA gene reads. After forming operational taxonomic units at 97 % identity, a total of 12,813 OTUs were recovered for the PSP samples and 12,032 OTUs for the MO BIO samples.

For the high biomass saliva samples, the major bacterial lineages were consistent between the two DNA purification methods. The saliva samples were high in *Streptococcus*, *Veillonella*, and *Prevotella*, paralleling many previous studies [11, 14, 19]. The lineages detected for samples purified with each of the two methods were identical at the OTU level.

Vaginal samples were collected as single swabs and thus could be assayed with only one DNA purification method. We chose PSP because it yielded particularly good DNA recovery from other types of microbiome samples [20]. Vaginal samples were high in *Lactobacillus*, as has been seen for vaginal samples in many studies [19, 21–23].

For the placental and background contamination samples, results diverged radically and were associated with the kit used to purify the DNA. For samples purified using the PSP kit, all placental samples and background controls contained *Sediminibacterium*, *Bradyrhizobiaceae*, *Methylobacterium*, and *Propionibacterium*. In most samples, the first three contributed the majority of the sequence reads. *Sediminibacterium*, from the family *Sphingobacteriaceae*, is a known soil bacterium. *Bradyrhizobiaceae* contains both soil- and plant-associated bacteria as well as animal-associated bacteria. *Methylobacterium* is a normal inhabitant of soil and water. *Propionibacterium* is a ubiquitous human skin organism that is common in house dust. All four lineages have all been reported to be common contaminants of DNA extraction kits [9, 10].

Additional lineages were prominent in a few samples. One of the maternal side biopsy samples was high in *Enterobacteraceae* and a second in *Prevotella*. Several contamination controls were high in *Phyllobacteriaceae*, another bacteria associated with soil and plants that has been identified as a contaminant of DNA extraction kits [9, 10].

For DNA purified from the MO BIO kit, the dominant lineage in the controls was an OTU aligning 100 % to *Clostridium difficile*. This OTU was predominant in 22 out of 23 samples, including fetal side and maternal side placenta, extraction blanks, unopened sterile swabs, and swabs exposed to air in the clinical laboratory. This OTU was found in a previous negative control sample from our laboratory in another study extracted with this kit, but not in negative control samples extracted with other kits, suggestive of contamination in commercial reagents. We have previously amplified *C. difficile* 16S rRNA gene sequences in our laboratory, so we cannot rule out that the low-level contamination originated from this source. However, as *C. difficile* only appeared in negative controls worked up with the MO BIO kit, we tentatively associate this contamination with the kit. Additionally, one sample was high in a lineage of the order *Streptophyta*, possibly a sequence derived from chloroplasts in pollen.

We attempted to investigate the origin of the sample-specific high proportion OTUs in the maternal side placental data set but were unable to specify the source. Of particular interest were connections with oral microbiota, since oral sites were proposed to donate lineages to placenta in the previous report on a commensal microbiome by Aagaard and colleagues [3]. For the PSP-extracted samples, although *Prevotella* lineages could be detected in subject 67’s oral samples, the OTU enriched in the maternal side placental biopsy was not among those present in the subject’s saliva sample, nor in her vaginal or fetal side placental samples. Thus, the data did not support the idea that the *Prevotella* detected in placenta originated at an oral site. For the *Enterobacteraceae* OTU enriched in the maternal side sample from subject 61, no reads were detected from this OTU in saliva or vaginal samples, and three reads were detected for fetal side placental samples (out of 7633). Thus, we were unable to trace the origin of these outlier lineages to specific body sites in our paired samples.

### Analysis of clustering by sample type

We next compared community structure by calculating distances between all pairs of samples and interrogating these data for clustering associated with sample type. In our first approach, we calculated Bray-Curtis distances for data pooled at the phylum level, paralleling the approach used by Aagaard et al. [3]. For each purification method, when all sample types were analyzed together, the difference among groups was highly significant (PERMANOVA *p* < 0.001 for comparison of the centroids of each group), driven by the formation of discrete clusters for the vaginal and oral samples. The analysis was then repeated, excluding the vaginal and saliva samples. In this case, PERMANOVA *p* values were >0.05 for PSP-extracted samples and 0.033 for MO BIO. The *p* value lower than 0.05 in the MO BIO analysis could be attributed to outliers in the unopened sterile swab controls—comparison of placental samples to swabs exposed to air in the clinical laboratory and extraction blanks only (dropping out the unopened swabs) showed a *p* value of 0.11. Thus, we conclude that we could not distinguish placental samples from contamination controls by PERMANOVA analysis of Bray-Curtis distances.

We repeated the PERMANOVA tests using weighted and unweighted UniFrac distances generated from operational taxonomic units (OTUs) clustered at 97 % sequence similarity, a more conventional approach (Additional file 4: Figure S3A-H). Once again, there was significant clustering by group when all groups were analyzed together (*p* = 0.001 for both weighted and unweighted UniFrac). When the vaginal and salivary samples were removed, *p* values were >0.05 for PSP-extracted samples and 0.024 and 0.049 for MO BIO (unweighted and weighted, respectively). Paralleling the results of the Bray-Curtis analysis, removal of the sterile swab data resulted in *p* values >0.05 for use of both weighted and unweighted UniFrac (comparing placental samples to air swabs and extraction blanks). Thus, we conclude that we could not distinguish placental samples from contamination controls using UniFrac and PERMANOVA, though we could readily distinguish oral and vaginal samples.

**Fig. 3:**
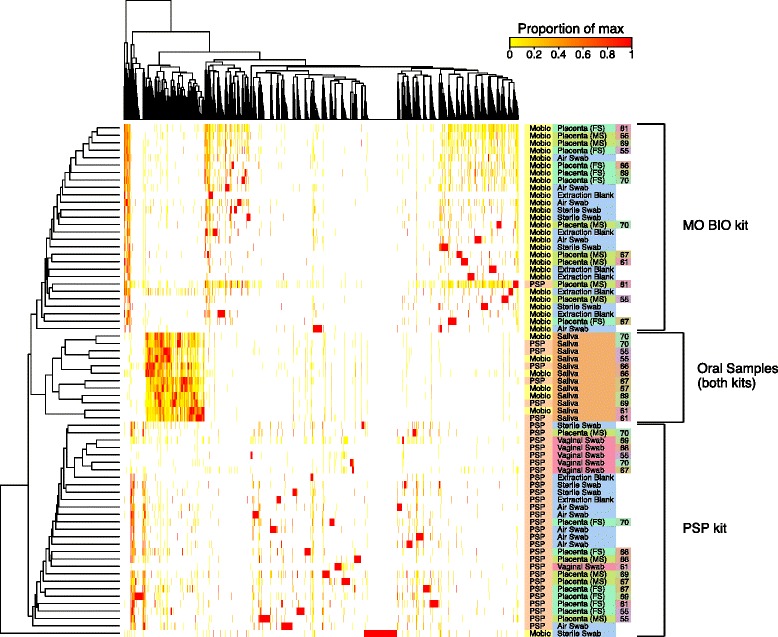
Analysis of microbial communities by co-occurrence. The heat map compares taxa observed within each sample, where each *row* represents a sample and each *column* an OTU. OTU sample combinations are colored to indicate abundance relative to the maximum proportion observed in any sample for that OTU. Thus, the *red cell* in each *column* indicates the sample with the highest proportional abundance for that OTU. Sample characteristics are given to the *right* of the plot and are color coded for convenience; *numbers* refer to the research subject ID. *Row* and *columns* are clustered by co-occurrence with trees indicating complete-linkage clustering of the Euclidean distance between the abundances of samples (*rows*) or OTUs (*columns*)

A heat map summarizing OTU representation in the sample set is shown in Fig. 3. Clustering by resemblance among samples pulled the oral samples together, with pairs of samples from each of the six individuals clustering together despite purification using different DNA purification kits. The placental samples and contamination controls were interspersed and mostly clustered by the kit used for purification (27/28 for MO BIO and 28/29 for PSP). The vaginal samples mostly clustered together (5/6) within the PSP group.

### Analysis of enriched lineages

We next asked whether any single lineages could be extracted from the data that might represent authentic placental microbiota. We reasoned that any lineages that were seen in placental samples extracted by both methods, but less abundant or absent in controls, would be candidates (though we note that there is evidence for occasional recovery of different lineages dependent on the DNA purification kit used [24]).

**Fig. 4:**
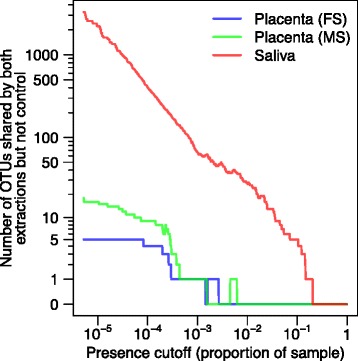
Enrichment of bacterial lineages detected reproducibly in placental or oral samples. The number of OTUs present in at least one saliva (*red*) or maternal (*green*) or fetal (*blue*) side fetal sample in both DNA extractions and not in negative controls at that abundance is shown over a range of abundance values (*x*-axis)

An overview of OTU sharing between pairs of samples extracted by the two methods is presented in Fig. 4, summarizing the community recovered over a wide range of abundance filters. Placental and salivary OTUs were first filtered by requiring recovery in both the PSP and MOBIO procedures, then the reproducible OTUs were compared to contamination. In this method, a reproducible OTU seen in placental or oral samples at a given proportion was counted if absent in contamination controls at or above that proportion. By this means, we scored the placental or oral OTUs enriched over contamination in comparisons spanning 5 logs of abundance values (Additional file 5: Table S3).

There were no placental OTUs that achieved at least 1 % abundance in both PSP and MOBIO in at least one sample and not in controls, In contrast, the same filtering procedure recovered 28 OTUs from salivary samples. Analyzing shared OTUs achieving 0.001 % in any sample, we found that 2197 OTUs were recovered in the oral samples, 16 in the maternal side placental samples, and 5 in the fetal side placental samples. Two OTUs appeared in both fetal and maternal side samples, leaving 19 unique placental OTUs. Of these OTUs, 16 OTUs were seen in one placenta only and three were seen in multiple placentas. The three OTUs found in multiple placentas could not be assigned to any bacterial taxa, and further analysis showed the sequences were 100 % identical to human chromosomal sequences. Thus, focusing on the more abundant OTUs, there were no OTUs that appear reproducibly in the placental samples with proportional abundance 1 % or higher that were not also present at the same or higher levels in the contamination controls.

## Discussion

Here, we compared bacterial DNA recovered from placental samples from six uncomplicated deliveries at term (both fetal side and maternal side internal tissues) to contamination controls. We assayed (1) the absolute number of 16S rRNA gene copies and (2) overall community structure queried by deep sequencing of 16S rRNA gene tags. In the qPCR analysis, both the placental samples and contamination controls yielded quite low values, with no clear signal in the placental samples above negative controls. In the community analysis, there was no clear separation of placental samples from contamination controls based on PERMANOVA analysis of the Bray-Curtis and UniFrac distances nor were any placental-enriched lineages detected reproducibly that were present as 1 % of the community or greater. Thus, we were unable to detect any consistent differences between the placental samples and contamination controls.

We compared two DNA purification methods, which helped to clarify the influence of contamination originating in the purification kits. Oral sites are known to host dense bacterial communities, and similar lineages were found in the oral samples regardless of the DNA purification method used. Placental and contamination control samples, in contrast, harbor little or no detectable bacterial DNA by QPCR; and for these samples, the bacterial lineages recovered were highly dependent on the purification method used. Thus, these data suggest that the predominant lineages observed in the placental and control samples originated as contamination that differed between kits. Recent control studies from others documented bacterial DNA contamination in DNA purification kits and differences among manufacturers, paralleling results here [9, 10].

We reasoned that authentic microbiota should be mostly reproducible regardless of the DNA extraction kit used and so focused on OTUs recovered in common using both extraction kits from placental or saliva samples (Fig. 4). In our analysis, we also required that the reproducible OTU be of greater proportional abundance in placenta or saliva than in any control. In the saliva samples, reproducible OTUs that met these criteria were readily detected. When we interrogated placental samples, we found no reproducible OTUs that were above 1 % in abundance and also at less than 1 % abundance in all contamination controls. At the lowest cut value, 0.001 % abundance, we found 18 OTUs reproducible between extractions for the maternal side sample and 5 for the fetal side sample. Some of these extremely low-abundance lineages may represent sporadic, rare placental microbiota. However, at this low level of abundance, noise is abundant in the data—several unclassified OTUs apparently arose from mispriming on human DNA. It is unlikely that we fully sampled all potential contaminants, and apparently enriched lineages could be found readily in comparisons between contamination data sets. Thus, an alternative explanation for the remaining placental OTUs is that they represent unsampled low-level contamination.

This study has several limitations. Our data do not rule out the existence of placental microbiota—we can only conclude that we could not use our data to distinguish placental samples from contamination controls. There could potentially be placental microbiota that is below our level of detection. We studied only six pregnancies, so it remains possible that with more subjects, a signal might be detected; though we would predict that detection would occur against a notable contamination background. The 16S rRNA gene window we used (V1V2) has biases in the lineages detected, as do all metagenomic methods, so any placental bacteria not detected by our amplification strategy would have been missed. We did not attempt to study the surface of the placenta due to likely issues with contamination during delivery—a recent paper studied placental microbiota specimens sampled near the surface of the placenta after vaginal delivery and found the swabs to be rich in lineages common in vagina [25]. If there is patchy microbial colonization, it might have gone undetected. Our data also have no bearing on the question of whether the placenta might be colonized in disease states because the six mother-baby pairs studied were healthy.

We can make several recommendations for future studies of placental microbiome samples and low biomass samples in general. (1) At the start of a study that might involve low biomass samples, it is essential to carry out some form of absolute abundance measure (here qPCR of total 16S rRNA gene copies) to determine whether samples of interest are indeed of low biomass, so that appropriate precautions can be taken. (2) When working with low biomass samples, start with the null hypothesis that all samples are contamination only and ask whether this idea can be rejected with data. (3) It is essential that contamination controls be generated and analyzed contemporaneously with experimental samples. Different DNA extraction kits are documented to have different contaminants, and contamination may vary by batch [9, 10]. Thus, comparison of unmatched samples and controls may lead to spurious distinctions. (4) In our hands, PCR amplification for library preparation sometimes does not yield detectable DNA by bulk measures such as gel electrophoresis, but low numbers of sequence reads can still be recovered—thus, it is important to sequence even libraries that appear to be failures in order to sample the contamination background fully. (5) Post hoc analysis, where the parent groups showed no global difference, can be risky. Ideally, results from any such analysis will be retested in an independent validation cohort [26]. (6) It would be helpful if reviewers of microbiome papers analyzing low biomass samples consistently asked authors to report contamination controls and their procedure for rejecting the hypothesis that all samples contain contamination only.

## Conclusions

We analyzed samples from oral, vaginal, and placental sites from six deliveries of healthy babies. Although we could readily detect distinctive microbial signatures in oral and vaginal samples, we were unable to distinguish placental samples from contamination controls using our data.

## Methods

### Human subjects studied

Patients were identified and enrolled as part of a large case–control study (Cellular Injury and Preterm Birth, CRIB) funded by the March of Dimes Prematurity Research Center at the University of Pennsylvania. All women in the current study were enrolled as term delivery controls in the CRIB study. Women were enrolled after admission to the labor and delivery unit at the Hospital of the University of Pennsylvania. The electronic medical records of potential study participants were screened by study coordinators to determine eligibility. Women aged 18–45 years with singleton pregnancies who were admitted to the hospital with spontaneous labor (regular contractions, cervical dilation) or premature rupture of membranes (PROM) and delivered at term (38^0/7^ to 41^6/7^ weeks gestational age) were eligible for enrollment as controls in the CRIB study. Exclusion criteria for controls were multiple gestation, fetal chromosomal abnormality, major fetal anomaly, intra-uterine fetal demise, intra-uterine growth restriction, gestational hypertension/preeclampsia, clinical chorioamnionitis, induction of labor, and elective cesarean delivery. To date, more than 100 women have been enrolled and samples collected for the CRIB study. We selected sample sets from six controls at random for the current study. The CRIB study has been approved by the Institutional Review Board at the University of Pennsylvania (protocol #821376).

### Sample collection protocol

Maternal saliva and vaginal samples were collected from participants after enrollment into the CRIB study. Placental samples were collected following delivery.

Maternal saliva samples were collected in sterile 50-mL conical tubes labeled with the participants’ study ID numbers. Each participant was asked to allow saliva to collect in her mouth for 1 min, after which she spit into the 50-mL tube. The tube was capped by the clinical research coordinator and placed immediately at −20 °C before transfer within 48 h to a −80 °C freezer for storage. In order to collect vaginal samples from study participants, trained clinical research coordinators performed a sterile speculum examination and inserted a labeled ESwab (Copan Diagnostics, Murrieta, CA) approximately 2 in. into the patient’s vagina, swirling the swab for 30 s making sure to contact the walls of the vagina so that the swab could absorb cervicovaginal fluid. ESwab is a liquid-based multipurpose collection and transport system that maintains viability of aerobic, anaerobic, and fastidious bacteria for up to 48 h at room and refrigerator temperature. The ESwabs were stored in collection tubes provided by the manufacturer at 4 °C and then moved to long-term storage at −20 °C.

Following delivery, placentas were placed in sterile containers on ice and transported by clinical research coordinators to the clinical laboratory located adjacent to the labor and delivery rooms at the Hospital of the University of Pennsylvania. Coordinators were trained by one of the principal investigators (SP) for the March of Dimes Prematurity Research Center at the University of Pennsylvania, and coordinators wore masks and sterile gloves during the placental dissections. A total of six 0.5 × 0.5 × 0.5 cm placental biopsies were obtained from each placenta—three from the maternal side of the placenta (basal plate), two from the fetal side, and one from the mid-placental region. Placental biopsies were obtained using established protocols that were modified slightly for the purposes of the CRIB study [3, 27, 28]. Before obtaining the placental biopsies, a sterile scalpel and forceps were used to take off the maternal surface of placenta (0.25–0.5-cm depth) in order to remove potential contaminants for microbiome studies. Biopsies were then obtained by sharp dissection from a region equidistant from the umbilical cord insertion site and the lateral edge of the placenta [27, 28]. Three maternal side biopsies (basal plate) were obtained first, then the mid-placental biopsy, and then the two fetal side biopsies. Each biopsy sample was rinsed in sterile phosphate-buffered saline, placed in a sterile, labeled cryovial, flash-frozen in liquid nitrogen, and stored at −80 °C. One maternal side biopsy sample and one fetal side biopsy sample were selected randomly for DNA purification by both of the methods for each of the six participants in the current study.

### DNA preparation

#### Method 1

DNA was isolated from placental biopsies, vaginal swabs, saliva, and controls using the PSP Stool DNA Plus kit (STRATEC Biomedical, Berlin-Buch, Germany) in a sterile class II laminar flow hood. In this sample set, air swabs of the clinical laboratory were matched to each patient and the associated samples. Swab tips were cut directly into bead tubes. The entirety of each tissue biopsy was placed into a tared bead tube and weighed (samples ranged from 0.11 to 0.48 g). One-hundred microliters of each saliva sample was used for DNA extraction. Lysing Matrix E tubes (MP Biomedical, Santa Ana, CA) and a bead beating protocol were used instead of the homogenization and pre-lysis procedure of the manufacturer’s protocol. Samples were beaten for 20 min with the PSP kit Stool DNA Stabilizer buffer using the TissueLyser II (Qiagen, Hilden, Germany). Samples were then incubated at 95 °C for 15 min, and DNA was extracted per manufacturer’s protocol. Extracted DNA was stored at −20 °C.

#### Method 2

DNA was isolated from replicate biopsies, saliva, and control samples using the MO BIO PowerSoil DNA Isolation Kit (MO BIO Laboratories, Carlsbad, CA) in a sterile class II laminar flow hood. Swab tips were cut directly into bead tubes. The entirety of each tissue biopsy was placed into a tared bead tube and weighed (samples ranged from 0.12 to 0.66 g). Seventy to 100 μL of each saliva sample was used for DNA extraction. Samples were incubated at 70 °C for 10 min and homogenized for 35 min on the TissueLyser II. DNA was then extracted per manufacturer’s protocol. Extracted DNA was stored at −20 ° C.

### Quantification of 16S rRNA gene copies using qPCR

Bacterial DNA abundance was quantified by quantitative PCR (qPCR) amplification of the V1V2 region of the 16S rRNA gene. qPCR reactions were performed in triplicate (25 μL each), using 1:2 dilutions of DNA template. Primer and probe sequences and amplification conditions are described in Additional file 6: Table S2 and [29, 30].

### PCR amplification of the VIV2 region of bacterial 16S rRNA gene for Illumina sequence analysis

For each sample, the 16S rRNA gene was amplified using Golay-barcoded universal primers 27F and 338R [31, 32], listed in Additional file 6: Table S2. PCR reactions were carried out in quadruplicate (25 μL each) with AccuPrime Taq DNA Polymerase High Fidelity (Thermo Fisher Scientific Inc, Waltham, MA) with the following recipe: 7.21 μL PCR-grade water, 2.5 μL 10× buffer II, 0.19 μL Taq, 5 μL each forward and reverse primer (2 μM), and 5 μL template DNA. PCR reactions were prepared in a PCR clean room. Reactions were run on an Applied Biosystems GeneAmp PCR System 9700 (Thermo Fisher Scientific Inc, Waltham, MA) with the following cycling conditions: initial denaturation at 95 °C for 5 min followed by 30 cycles of denaturation at 95 °C for 30 s, annealing at 56 °C for 30 s, and extension at 72 °C for 90 s, with a final extension of 8 min at 72 °C. Replicate amplicons were pooled and bead purified using Agencourt AMPure XP (Beckman Coulter, Indianapolis, IN) with the manufacturer’s protocol. Reaction products were sequenced using the Illumina MiSeq technology [33].

### 16S rRNA gene sequence analysis

The 16S rRNA gene reads were analyzed using the QIIME software package [34] with default parameters augmented by the package qiimer (github.com/kylebittinger/qiimer) and the R programming language [35, 36]. Reads were removed from the analysis if they did not match a 12-base Golay barcode with 1 or fewer errors, if the paired reads failed to overlap by 35 bases, if the overlapped region differed by more than 15 %, or if they had more than 3 base calls below Q20. Operational taxonomic units (OTUs) were created by clustering the reads at 97 % identity using UCLUST [37]. Representative sequences from each OTU were aligned using PyNAST [38], and a phylogenetic tree was inferred using FastTree v. 2.1.3 [38, 39] after applying the standard Lane mask for 16S rRNA gene sequences [40]. Pairwise UniFrac distances were computed using QIIME [41], and permutational tests of distance were performed using the vegan library for the R programming language [42]. Principal coordinates analyses were performed with the APE library for R [43]. Taxonomic assignments were generated by the UCLUST consensus method of QIIME 1.8 [44], using the GreenGenes 16S rRNA gene database v. 13_8 [45]. Bayesian analysis of qPCR data was performed using the R library BEST [46].

## Additional files

Additional file 1: Table S1. Demographic and clinical characteristics of study participants. (DOC 29 kb) Additional file 2: Figure S1. Raw data from quantitative PCR analysis of 16S rRNA gene. (A–C) Representative amplification curves for 16S rRNA gene qPCR of placental and control samples in triplicate in the six subjects studied. (PDF 4964 kb) Additional file 3: Figure S2. Posterior probability distributions for the difference in DNA abundance between air swab negative controls and placenta (blue), saliva (red), vaginal swabs (orange), and other negative controls (green) as estimated by quantitative PCR analysis of 16S rRNA gene abundance for PSP (top) and MO BIO extractions (bottom). Each cycle difference in the cycle of threshold between samples was assumed to represent a 2-fold difference in DNA. Dashed vertical line indicates no change in 16S rRNA gene abundance compared to air swabs. (PDF 239 kb) Additional file 4: Figure S3. Analysis of microbiota composition using principal coordinate analysis of UniFrac distances. (A, E) Weighted UniFrac analysis of the full sample set. (B, F) Unweighted UniFrac analysis of the full sample set. (C, G) Weighted UniFrac analysis of placental and control samples only. (D, H) Unweighted UniFrac analysis of placental and control samples only. A–D corresponds to the PSP extraction method and E–H corresponds to the MO BIO extraction method. (PDF 288 kb) Additional file 5: Table S3. Lineages detected reproducibly in placental samples and their proportional abundance. (DOC 47 kb) Additional file 6: Table S2. Oligonucleotides used in this study. (DOC 34 kb)
