# Supplementary material for: Comparison of placenta samples with contamination controls does not provide evidence for a distinct placenta microbiota
Source: Microbiome. 2016 Jun 23;4:29. doi: 10.1186/s40168-016-0172-3 (PMC4917942; doi:10.1186/s40168-016-0172-3)
Supplement: Additional file 1: Table S1. — Demographic and clinical characteristics of study participants. (DOC 29 kb) [file 40168_2016_172_MOESM1_ESM.doc]

Additional file 1: Table S1. Demographic and clinical characteristics of study participants.

| **Patient ID** | **Maternal age, yr** | **Race** | **Gest. age at delivery, wk** | **Nulli-parous** | **Mode of delivery** | **Clinical presentation** | **Intra-partum anti-biotics** |
| --- | --- | --- | --- | --- | --- | --- | --- |
| 55 | 30 | Caucasian | 39 | Yes | SVD | Labor | No |
| 61 | 27 | African-American | 39 | No | C-section (early labor) | Labor | No |
| 66 | 22 | African-American | 41 | No | SVD | Labor | No |
| 67 | 28 | Asian | 38 | No | SVD | Labor | No |
| 69 | 40 | Asian | 40 | No | SVD | PROM | No |
| 70 | 25 | Caucasian | 40 | Yes | SVD | PROM | No |

SVD = spontaneous vaginal delivery

PROM = premature rupture of membranes (preceding labor)
