# Supplementary figures and images for: Comparison of placenta samples with contamination controls does not provide evidence for a distinct placenta microbiota
Source: Microbiome. 2016 Jun 23;4:29. doi: 10.1186/s40168-016-0172-3 (PMC4917942; doi:10.1186/s40168-016-0172-3)

**Figure S1**

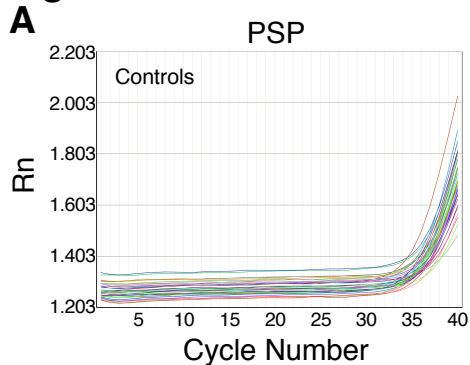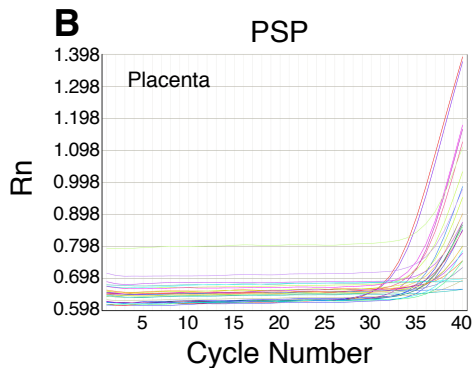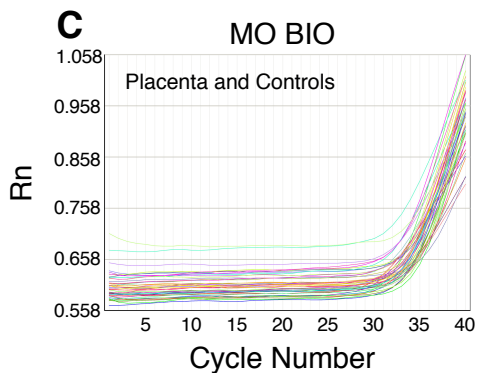

Supplement: Additional file 2: Figure S1. — Raw data from quantitative PCR analysis of 16S rRNA gene. (A–C) Representative amplification curves for 16S rRNA gene qPCR of placental and control samples in triplicate in the six subjects studied. (PDF 4964 kb) [file 40168_2016_172_MOESM2_ESM.pdf]

**Figure S2**

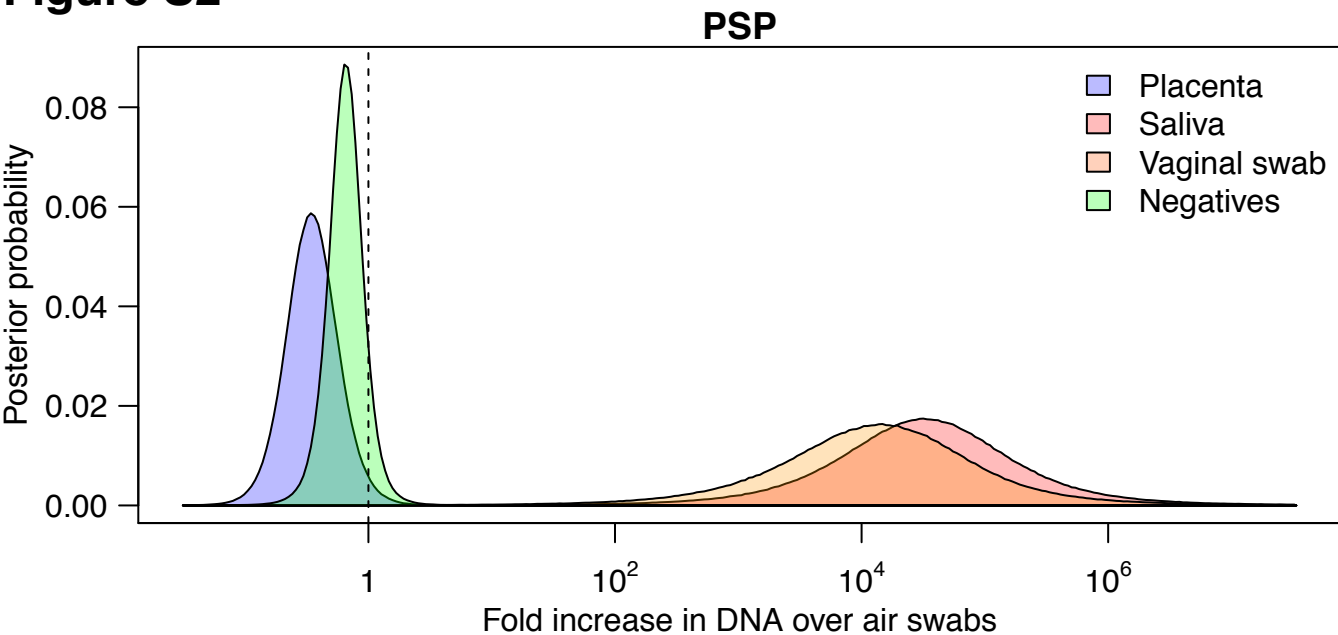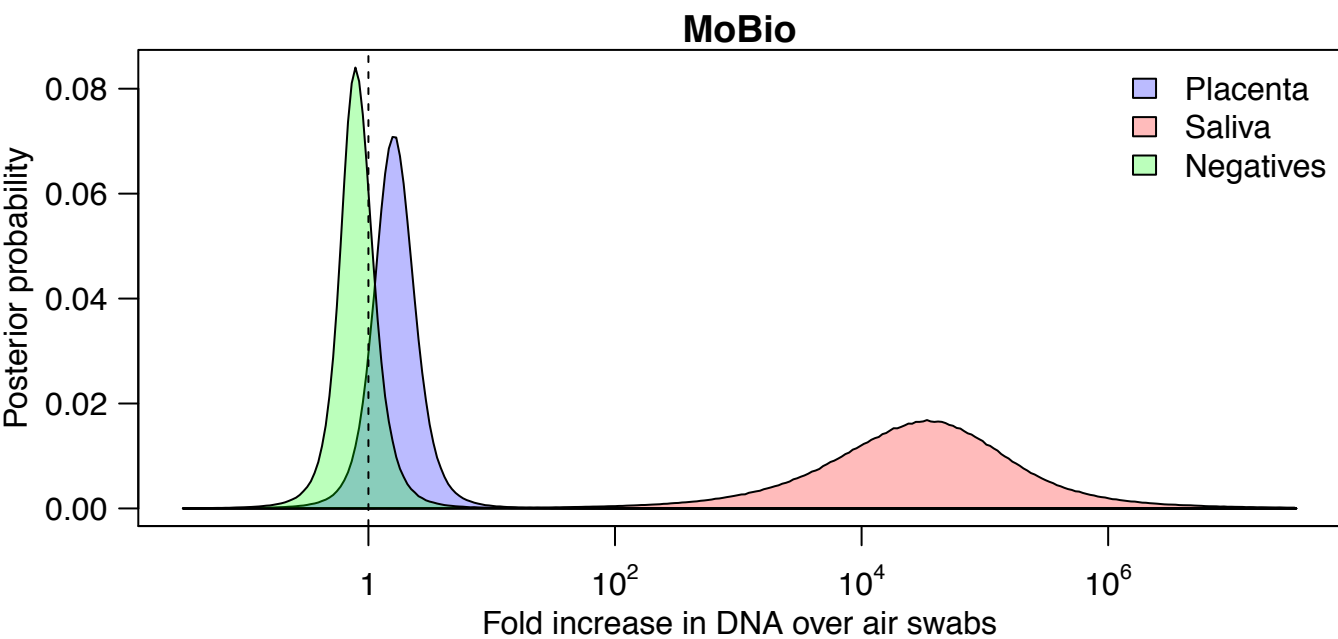

Supplement: Additional file 3: Figure S2. — Posterior probability distributions for the difference in DNA abundance between air swab negative controls and placenta (blue), saliva (red), vaginal swabs (orange), and other negative controls (green) as estimated by quantitative PCR analysis of 16S rRNA gene abundance for PSP (top) and MO BIO extractions (bottom). Each cycle difference in the cycle of threshold between samples was assumed to represent a 2-fold difference in DNA. Dashed vertical line indicates no change in 16S rRNA gene abundance compared to air swabs. (PDF 239 kb) [file 40168_2016_172_MOESM3_ESM.pdf]
