# Supplementary material for: Comparison of placenta samples with contamination controls does not provide evidence for a distinct placenta microbiota
Source: Microbiome. 2016 Jun 23;4:29. doi: 10.1186/s40168-016-0172-3 (PMC4917942; doi:10.1186/s40168-016-0172-3)
Supplement: Additional file 4: Figure S3. — Analysis of microbiota composition using principal coordinate analysis of UniFrac distances. (A, E) Weighted UniFrac analysis of the full sample set. (B, F) Unweighted UniFrac analysis of the full sample set. (C, G) Weighted UniFrac analysis of placental and control samples only. (D, H) Unweighted UniFrac analysis of placental and control samples only. A–D corresponds to the PSP extraction method and E–H corresponds to the MO BIO extraction method. (PDF 288 kb) [file 40168_2016_172_MOESM4_ESM.pdf]

Figure S3

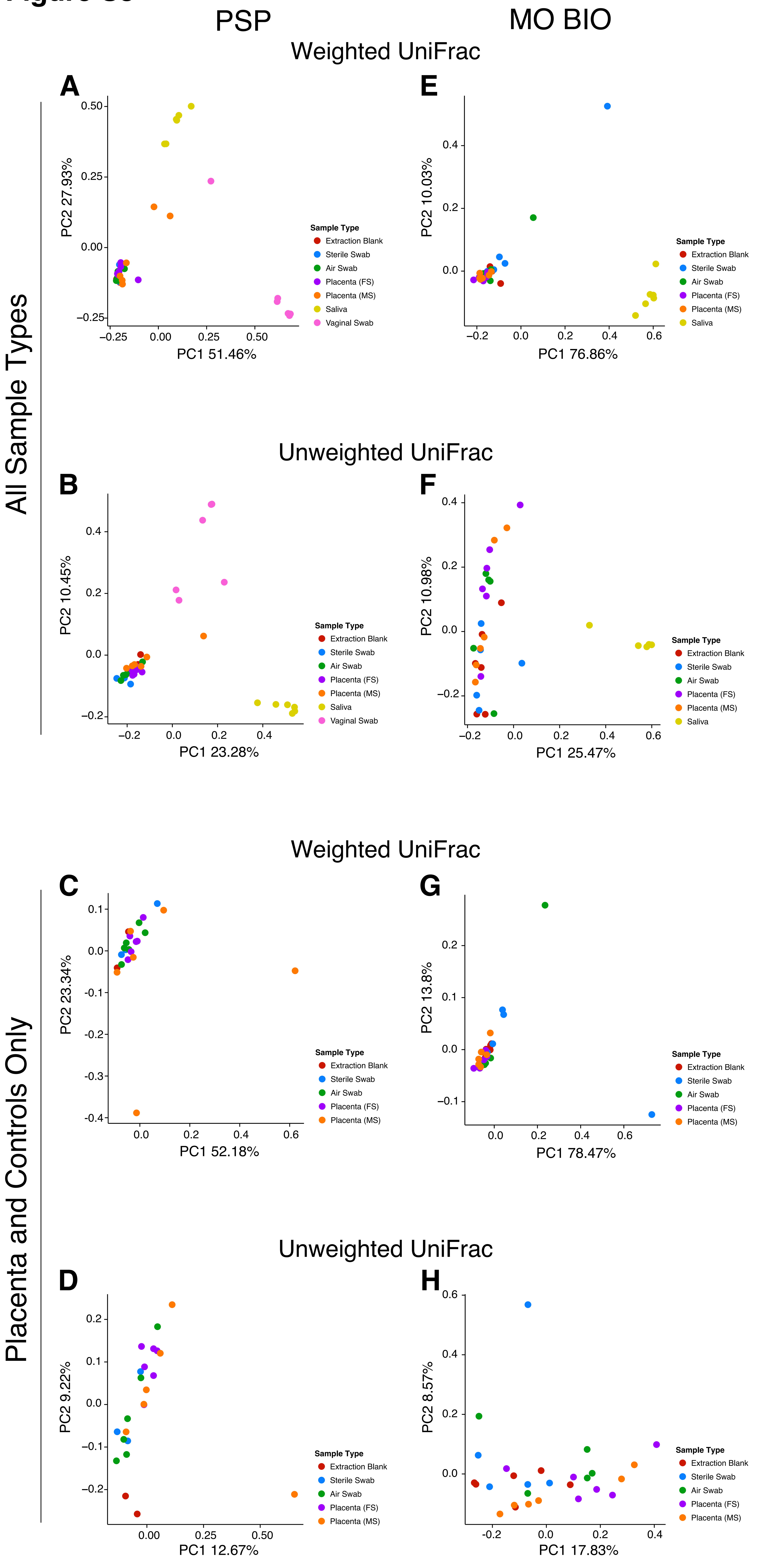

PSP
MO BIO
All Sample Types
Placenta and Controls Only
Weighted UniFrac
A
PC1 51.46%
PC2 27.93%
Sample Type
Extraction Blank
Sterile Swab
Air Swab
Placenta (FS)
Placenta (MS)
Saliva
Vaginal Swab
E
PC1 76.86%
PC2 10.03%
Unweighted UniFrac
B
PC1 23.28%
PC2 10.45%
F
PC1 25.47%
PC2 10.98%
Weighted UniFrac
C
PC1 52.18%
PC2 23.34%
G
PC1 78.47%
PC2 13.8%
Unweighted UniFrac
D
PC1 12.67%
PC2 9.22%
H
PC1 17.83%
PC2 8.57%
