# Supplementary material for: Comparison of placenta samples with contamination controls does not provide evidence for a distinct placenta microbiota
Source: Microbiome. 2016 Jun 23;4:29. doi: 10.1186/s40168-016-0172-3 (PMC4917942; doi:10.1186/s40168-016-0172-3)
Supplement: Additional file 5: Table S3. — Lineages detected reproducibly in placental samples and their proportional abundance. (DOC 47 kb) [file 40168_2016_172_MOESM5_ESM.doc]

Additional file 5: Table S3. Lineages detected reproducibly in placental samples, and their proportional abundance.

| Supplementary Table 3. OTUs found in placenta but not controls, and their proportional abundance. | | | | | |
| --- | --- | --- | --- | --- | --- |
| ID | Taxa | Placenta (FS) | Placenta (MS) | PSP controls | MO BIO controls |
| denovo15726 | Unassigned #1176 | 0.00145518 | 0.00043611 | 0 | 0 |
| denovo2235 | g__Sphingobium #1 | 0.000297089 | 0 | 0 | 0 |
| denovo15724 | g__Bacteroides #6 | 0.000263644 | 0 | 0 | 0 |
| denovo1582 | g__Streptococcus s__anginosus #1 | 0.000197733 | 0 | 0 | 0 |
| denovo7082 | Unassigned #536 | 8.25E-05 | 0.000279681 | 0 | 0 |
| denovo102 | Unassigned #11 | 0 | 2.64E-05 | 0 | 0 |
| denovo2327 | Unassigned #187 | 0 | 0.000144321 | 0 | 0 |
| denovo3923 | g__Lactobacillus #294 | 0 | 0.000198854 | 0 | 0 |
| denovo4048 | o__Clostridiales #177 | 0 | 0.000376776 | 0 | 0 |
| denovo7497 | o__Clostridiales #334 | 0 | 3.14E-05 | 0 | 0 |
| denovo9452 | o__Bacteroidales #32 | 0 | 7.33E-05 | 0 | 0 |
| denovo9572 | g__Candidatus Aquiluna s__rubra #1 | 0 | 0.00030011 | 0 | 0 |
| denovo12339 | Unassigned #918 | 0 | 0.000288642 | 0 | 0 |
| denovo13040 | Unassigned #983 | 0 | 5.45E-05 | 0 | 0 |
| denovo13204 | g__Prevotella s__stercorea #10 | 0 | 3.66E-05 | 0 | 0 |
| denovo13650 | f__Enterobacteriaceae #197 | 0 | 5.23E-06 | 0 | 0 |
| denovo14191 | o__Bacteroidales #58 | 0 | 1.05E-05 | 0 | 0 |
| denovo14239 | f__[Mogibacteriaceae] #32 | 0 | 0.000204087 | 0 | 0 |
| denovo14596 | g__Blautia #24 | 0 | 1.57E-05 | 0 | 0 |
| denovo15381 | g__Peptococcus #6 | 0 | 0.001540436 | 0 | 0 |
| denovo17692 | Unassigned #1327 | 0 | 5.23E-06 | 0 | 0 |

**List of Abbreviations**

1. MO BIO is the abbreviated term used for the MO BIO PowerSoil DNA Isolation Kit, one of the extraction methods used in this study.
2. PSP is the abbreviated term used for the STRATEC PSP Spin Stool DNA Plus Kit, the second extraction method used in this study.
