# Supplementary material for: Comparison of placenta samples with contamination controls does not provide evidence for a distinct placenta microbiota
Source: Microbiome. 2016 Jun 23;4:29. doi: 10.1186/s40168-016-0172-3 (PMC4917942; doi:10.1186/s40168-016-0172-3)
Supplement: Additional file 6: Table S2. — Oligonucleotides used in this study. (DOC 34 kb) [file 40168_2016_172_MOESM6_ESM.doc]

Additional file 6: Table S2. Oligonucleotides used in this study.

|  | **Oligo Name** | **Sequence** | **Components** |
| --- | --- | --- | --- |
| (A) Illumina MiSeq 16S V1V2 Library Preparation & Sequencing Primers | Forward PCR Amp Primer | 5'-AATGATACGGCGACCACCGAGATCTACAC-ACGAGACTGATT-TATGGTAATT-GT-AGAGTTTGATCCTGGCTCAG-3' | 5' Illumina adapter, 12-base Golay barcode (example), forward pad*, linker**, 27F forward primer |
|  | Reverse PCR Amp Primer | 5'-CAAGCAGAAGACGGCATACGAGAT-TCCCTTGTCTCC-AGTCAGTCAG-CC-TGCTGCCTCCCGTAGGAGT-3' | Reverse complement of 3' Illumina adapter, 12-base Golay barcode (example), reverse pad, linker, 338R reverse primer |
|  | Forward Seq Primer | 5'-TATGGTAATT-GT-AGAGTTTGATCCTGGCTCAG-3' | Forward pad, linker, 27F forward primer |
|  | Reverse Seq Primer | 5'-AGTCAGTCAG-CC-TGCTGCCTCCCGTAGGAGT-3' | Reverse pad, linker, 338R reverse primer |
|  | Reverse Barcode Seq Primer*** | 5'-ACTCCTACGGGAGGCAGCA-GG-CTGACTGACT-3' | Reverse complement of 338R reverse primer, linker, reverse complement of reverse pad |
| (B) 16S V1V2 qPCR Oligos | Forward qPCR primer | 5’-AGAGTTTGATCCTGGCTCAG-3’ | BSF8 primer |
|  | Reverse qPCR primer | 5´-TCGACTTGCATGTRTTA-3´ | BSR65/17 primer |
|  | qPCR probe | 5' - /56-FAM/TAA +CA+C ATG +CA+A GT+C GA/3BHQ_1/ - 3' | Fluorescent dye (5' 6-FAM (Fluorescein)), landing sequence, dark quencher (3' Black Hole Quencher®-1)  *A + indicates a locked nucleic acid base. |

Oligonucleotide sequences used for (A) 16S rRNA gene library preparation and sequencing on the Illumina MiSeq platform and (B) 16S rRNA gene qPCR. (A) In a single PCR reaction, the 16S rRNA gene region of a sample is amplified and tagged with a unique Golay barcode combination, and Illumina adaptor sequences are appended to each end of the amplicon. *Forward and reverse pad sequences increase primer melting temperatures. **Linker sequences separate pad and 16S rRNA gene-specific primer sequences. ***Forward barcode sequencing primers need not be added as Illumina flow cell-bound adapter oligonucleotides prime this sequencing reaction. (B) *The qPCR probe sequence is slightly modified from the original design ; locked nucleic acids were added to increase melting temperature. All oligonucleotides were purchased from Integrated DNA Technologies.
